# Medical News and Items

**Published:** 1876-02

**Authors:** 


### Medical News and Items.

Library Opening.—After an unavoidable delay, the library of the Chicago Medical Press Association was formally inaugurated on the evening of the 30th ult.

A special invitation had been sent by the librarian to each member of the Association, and a general invitation had been extended to the profession at large ; in response to which, some sixty representative members of the profession met in the library rooms, at 263 Wabash Avenue.

An address of welcome to the new library was given by the President of the Association, Dr. R. C. Hamill. The librarian was next called upon, and briefly stated the object of the meeting. The feature of the evening which had been specially announced, was a salutatory by Dr. Jas. Nevins Hyde, who then delivered a humorous speech in the Latin language. Notwithstanding there were many who regretted that their "Stoddard" or "Harkness" had been allowed to grow dusty and rusty, yet the effect of the speech was most happy, and was the signal for a social interchange of thought and sentiment which was not lost sight of.

Speeches were made and remarks offered upon invitation, by Drs. N. S. Davis, J. P. Ross, H. A. Johnson, W. T. Byford, A. R. Jackson, J. H. Etheridge, J. Bartlett, N. Bridge, A. Fisher, and by the "secular editor," Mr. Cooke, of Keen, Cooke & Co. The report of the treasurer was also called for and read by Dr. J. E. Owens.

---

Our County Hospital for the Insane is a particularly interesting object for study to every man who desires to know how the insane ought *not* to be cared for. The mismanagement of the insane at the County Asylum has repeatedly been commented upon by the secular press, though without avail. In November, the

Chicago Society of Physicians and Surgeons appointed a committee to investigate into this matter. And at the last meeting of this society, held on January 24, this committee was ready to report on the result of their labor. The lengthy report was listened to by some sixty physicians with the greatest attention, for it contains a great many interesting facts, and is filled with observations which well serve to convince everybody of the necessity of an humane society.

It will enlighten our readers to learn the modus operandi by which the charitable institutions of Cook County are managed, and therefore the following items, clipped from the report of the medical experts, may be interesting:

"Several years ago, when preparations were being made for the erection of the first or northern portion of the present building for the insane, certain competent medical men were consulted as to the choice of a site, and the present one was strongly disapproved of and another selected. But, *in spite of these recommendations, the asylum was placed where it now is.*

The location is one of the dreariest, flattest, most exposed in the country, and on many accounts unsuited for its purpose.

The asylum is under the control of the Board of County Commissioners, and they are finally responsible for its condition and management. Upon these commissioners devolves the duty of appointing the officers of the hospital, and among them the physicians.

Besides the physicians, there is a warden, who, in accordance with the rules for the government of the asylum, drawn up by the commissioners, is the superior officer. The particular rule in question reads as follows:

RULE 5. Every other officer or agent for the county at the insane asylum or poor-house must hold himself subordinate to the warden, as the warden will be held accountable for the proper management of the institution.

The physician is elected yearly by the commissioners, and this year for the first time, as far as we are informed, has an assistant physician been elected.

There is no regular apothecary, as at all State Asylums.

One of these physicians is known to one member of the committee to be an excellent young man who has attended one course of lectures in one of the colleges in this city, and who went to the asylum some time since, hoping to make his sojourn profitable in the way of study. He has never completed his ordinary studies, and has never, until since his residence of a year or so at that hospital, had any experience of any kind in the treatment of disease, worthy of the name. He is in no sense qualified to assume the position of physician to such an institution, and he does not pretend to be so qualified, and yet he has been recently elected on a salary as assistant physician to the asylum.

As regards the superintendent, he is also a young man, who, from the best we can learn, cannot have had an extensive experience in the treatment even of common forms of disease, and certainly not in the management of the insane. The committee have had some reason to doubt whether he is even a graduate in medicine.

They know that he has placed himself on record in this city as a graduate of the Bellevue Hospital Medical College of New York City, and that he has so represented himself to more than one member of the profession in this city. Yet on inquiring of the secretary of Bellevue Hospital Medical College, the committee received the answer that no such name appeared on the books either as a student or graduate of that college.

We have no fear of being successfully contradicted when we say that the supply of food at our county asylum is neither so good as the insane require, nor as the cost of maintaining our insane should afford them. At the time of a former visit of the chairman of the committee on his own responsibility, he found the beds, especially in the lower wards, very coarse and uncomfortable, and destitute of pillows. At the time of the visit of the committee, the same kind of beds, consisting of ticks filled with coarse straw, were in use, but they were supplied with straw pillows, stuffed with similar material with the ticks themselves.

We do not consider them such as should be provided for the insane, certainly far below what are provided in our State hospitals, and for the same or less money. Neither did the clothing of the patients, at least of many of them, appear to the committee to be as good as furnished at Elgin and Jacksonville. It certainly did not seem to be as good as it should be for the money that these patients cost the county.

We have intimated that the accommodations should be better than they are, considering their cost. The asylum costs the county annually about $70,000 in round numbers. This includes simply the total sum expended, in the case of the insane, in supplying food, clothing, medicines, salaries of officers, attendants, etc. We have placed the average number of insane persons at the asylum at 300. But the truth is, 275 would be nearer the average for the past year. This would make the cost of maintaining patients, exclusive of the use of the building, which does not enter into the calculation, about $4.50 to $5 per week. This sum furnishes thousands of boarders in this city with better accommodations, and at the hands of private parties, who expect a margin, as profit on this price.

In the State hospitals of Massachusetts, with the exception of that of Tewksbury, the expense of State patients is $3.50 per week. None of these are specially designed for the pauper insane, but are rather of the character of our own State institutions, and the provision, except in the matter of buildings, is much above that for our insane paupers at Jefferson ; and, curiously enough, the cost is less. The pauper insane of Massachusetts are comparatively well cared for at Tewksbury,

*at a cost of* $2 *per week.* This institution, is under the most competent medical management.

Much the same statement is to be made concerning the asylum at Cranston, in Rhode Island.

In nearly all these cases the insane are cared for at as little or much less expense, and with better fare, than at Jefferson. We call your especial attention to this fact.

We have also compared the cost of maintaining our insane hospital for paupers with two Western institutions, viz., the Northern hospital at Oshkosh, Wis., and the Northern hospital at Elgin, both of which are State institutions. At Oshkosh, the patients are furnished in a very superior manner, with spring beds and hair mattresses on good large bedsteads, and other needful furniture, with excellent food, and first-class medical service—in all these respects better than at our own asylum, and for $4.50 a week per patient."

And so it goes on. Bad location, incompetent physicians, poor food, poor beds and clothing, and all this for $70,000 per annum. *Sapienti sat!*

---

The Iowa Central Medical Association held its third annual meeting Jan. 11, 1876, at which the following officers were elected, viz.:

President—Dr. Enoch Lewis, of Albion.

Vice-President—Dr. W. A. Chapman, of Marshalltown.

Secretary and Treasurer—Dr. Charles Reiterman, of Le Grand.

The annual address by Dr. O. F. Hixson, the retiring president, on the general subject of medicine and the profession, was a very able one. The next quarterly meeting will be held on the 11th of April.

---

Evening Lectures.—Arrangements have been completed for a course of lectures to be given to the members of the dental profession, on Thursdays and Saturdays from 7 to 9 P. M. These lectures embrace the subjects of Physiology, Histology, Pathology and Chemistry ; they began on the first Thursday in January, to continue for thirteen weeks. The inaugural lecture on "The Present Tendency of Science," was delivered by Dr. I. N. Danforth, on January 3rd.

---

THE ADVERTISEMENTS of the *N. Y. Medical Journal*, published by its exchanges, contain the encomiums of three British and three American periodicals. Of the latter, one is taken from the *Chicago Medical Times*—the small organ of the still smaller, so-called, Eclectic school of medicine in this city. It is now in order for the *N. Y. Medical Journal* to publish its opinion of the *Chicago Medical Times*. This advertisement of the two periodicals is gratuitous.

---

DR. CALVIN ELLIS, of Boston, has an article in a late number of the *Boston Medical Journal*, the object of which is, to impress on the professional mind that there is no such thing as *general* softening of the brain, clinically considered. He quotes from half a dozen or more authors whose phraseology implies that there is such a disorder as general softening, although no one positively says so. It is presumed no one would say so, yet with them, the profession in general are in the habit of talking and writing as though all cases of cerebral softening were not of a part, and usually a small part, instead of the whole of the brain mass.

There is little doubt that many of our beliefs, right and wrong, are the offspring of the phraseology we habitually —automatically—use, and Dr. Ellis has done a good service in jogging our memories on the subject of one abuse in this direction.

PROF. DEPAUL, of Paris, has lately returned from Brazil, whither he went at the instance of the Emperor to attend his daughter, the Countess d'Eu, in her recent confinement. It seems the Brazilian doctors are no more perfect than those of America. The reception and treatment of Prof. D. was very cold and uncordial until after the successful termination of the accouchement, which had been difficult, when all classes, the profession included, fêted him enthusiastically.

---

THE PROFESSION in Canada are in trouble about the laws regulating the practice of medicine. While the regular profession are discussing what sort of a bill they shall ask the legislature to enact, to better their condition, and are holding meetings for this purpose, behold a bill "which no person had ever seen or even heard about" is introduced into the House. The *Canada Journal* is suspicious of the bill, and we think with reason, for the document fills twelve brevier pages of the *Journal*, and was drawn by a lawyer. We fear if this bill passes, the profession in Canada will have no time for scientific pursuits.

---

THE IMPERIAL BOARD OF HEALTH IN GERMANY.—A sum amounting to about $4,000 has been appropriated to the establishment of a Supreme Board of Health for the German empire, the principal object of which will be to collect reliable statistics in sanitary matters, although it is reported that the board is also to be a sort of sanitary court of appeals, to which the district administrative boards or provincial governments will have to refer for sanitary advice and decision.

---

LEECHES PRESERVED IN SALICYLIC ACID.—The *France Médicale* contains the following: In a saturated solution of salicylic acid the leeches die very quickly, but in a solution containing just a little acid, they keep better than in common water. In a mixture of 100 grammes of water with four drops of a watery solution of salicylic acid (1.300), leeches can be kept for use during many months, without the water acquiring an offensive odor.

---

An Agreeable Cathartic.—Dr. P. Reich finds a "tinctura rhamni frangulæ" (buckthorn) an agreeable form of administration of the cathartic, and one that is particularly appropriate to cases in which it has to be given for a prolonged period. The usual dose of his tincture is two teaspoonsful at night, but in some cases one suffices, causing a pultaceous stool the following morning.—*Philad. Med. Times.*

---

The Report of the Rudolph Hospital at Vienna, for the year 1874, shows that during that year, 5,575 patients were received and 479 were transferred from 1873; making a total of 6,254 patients. Discharged cured, 3,375; improved, 1,298; incurable, 346; died, 653; remaining at the end of the year, 582.

---

*Embarras des richesses!* During the past two months our medical societies transacted so much important and interesting business that their reporters seem to be completely overwhelmed and unable to give us a fair record of these transactions. We make this statement lest some of our readers, not finding the usual society reports in this Journal, should believe the societies defunct.

